# Supplementary material for: An Integrative Analysis of Nasopharyngeal Carcinoma Genomes Unraveled Unique Processes Driving a Viral-Positive Cancer
Source: Cancers (Basel). 2023 Feb 15;15(4):1243. doi: 10.3390/cancers15041243 (PMC9953764; doi:10.3390/cancers15041243)
Supplement: Supplementary file 1 [file cancers-15-01243-s001.zip › cancers-2190189 - Supplementary Figures-done.pdf]

cancers

MDPI

Article

An Integrative Analysis of Nasopharyngeal Carcinoma Genomes Unraveled Unique Processes Driving a Viral-Positive Cancer

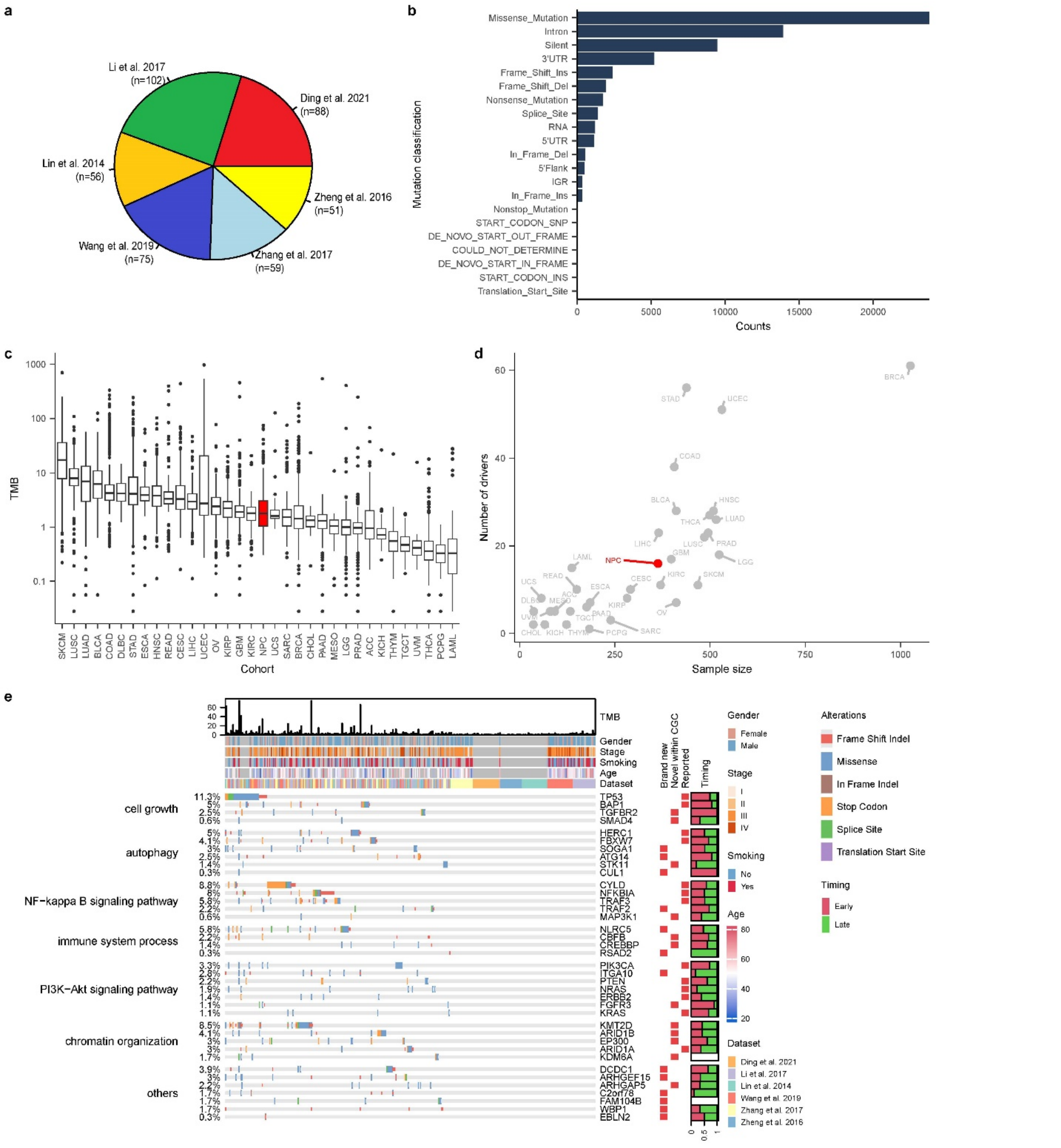

Figure S1. The genomic landscape of NPC. a) The cohorts and their sample sizes collected for this study, b) The mutation types and counts of the somatic mutations. c) The boxplot of TMB for the NPC as well as the TCGA cohort. d) The sample size (x-axis) vs number of driver genes identified (y-axis) relationship. e) Oncoprint plot at the pathway levels (similar to Figure 1 in the maintext).

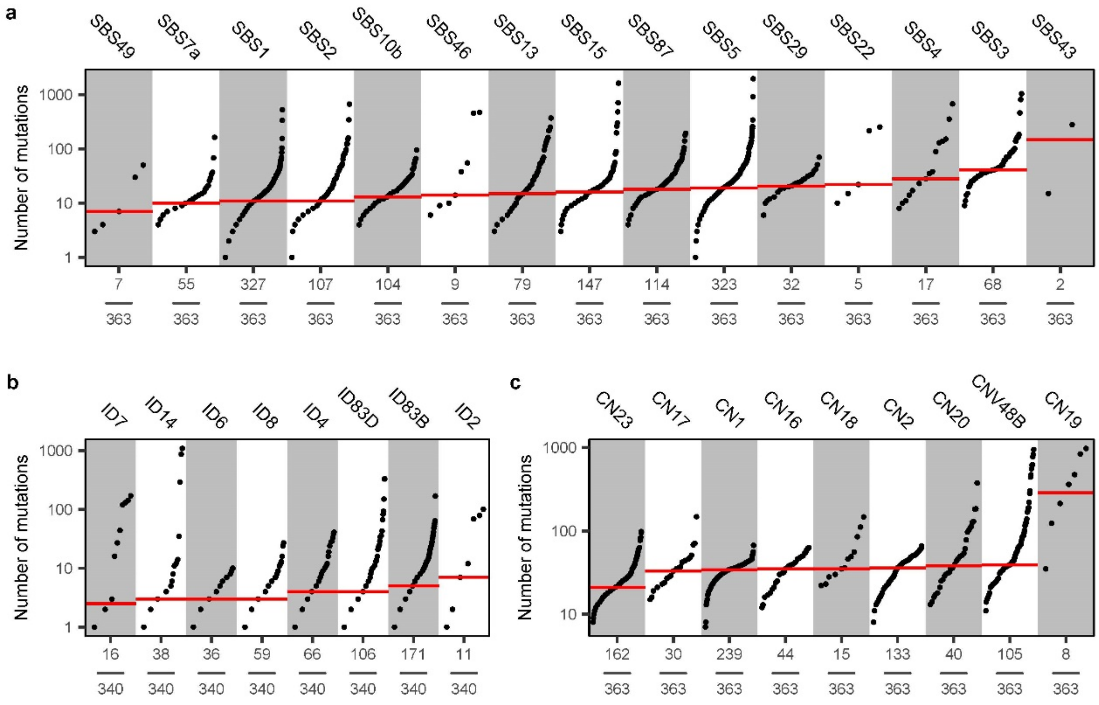

Figure S2. Signature contributions across patients. a) Signature contributions (TMB) of different SBS signatures. The numbers on the x-axis are the number of patients carrying the signature out of 363 patients. b) Signature contributions of different indel signatures. The numbers on the x-axis are the number of patients carrying the signature out of 340 patients. c) Signature contributions of different CNV signatures. The numbers on the x-axis are the number of patients carrying the signature out of 363 patients.

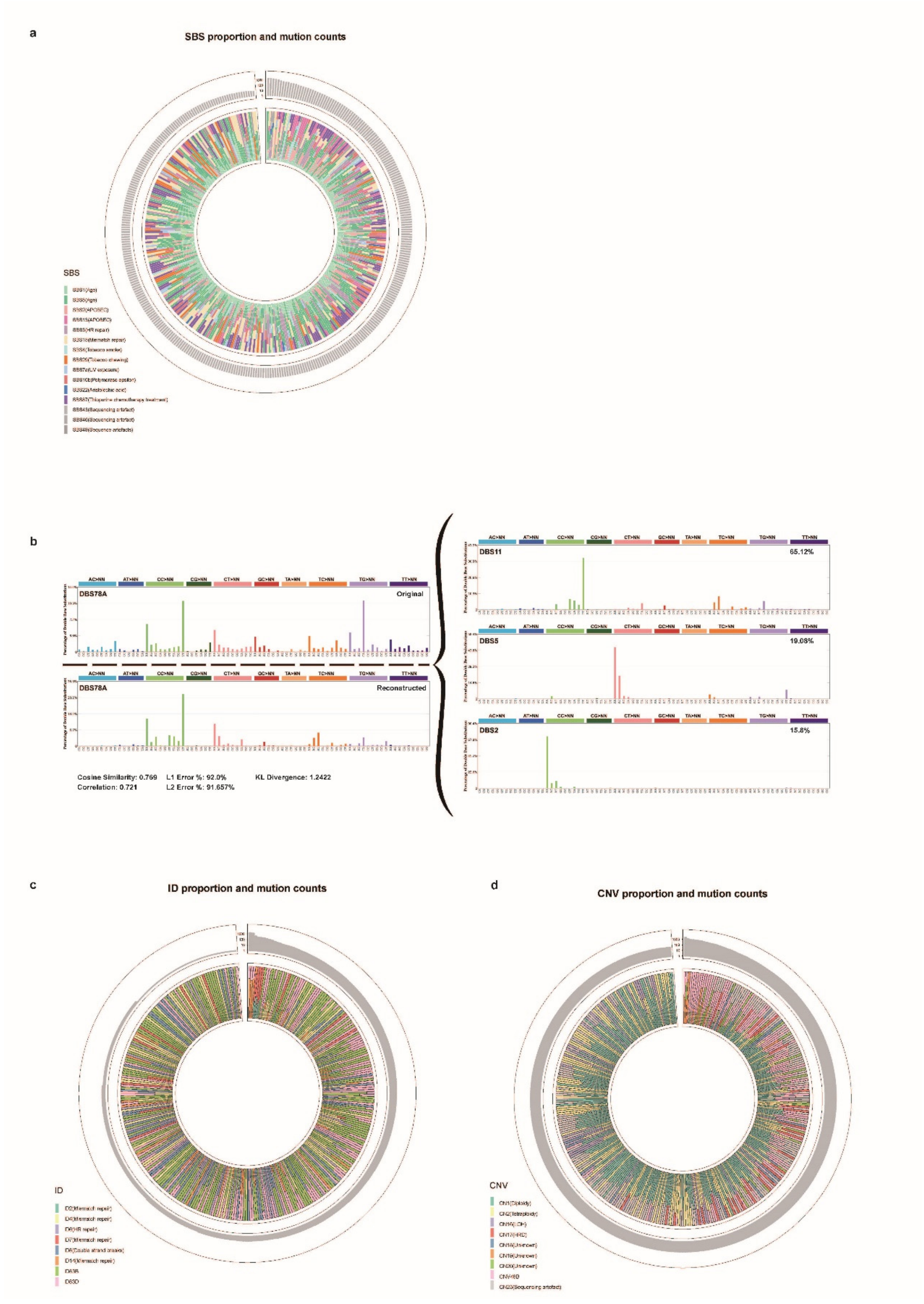

Figure S3. The contribution of different signatures across the cohort. a) Circus plot of the contributions of SBS signatures. The outer circle plots the number of single base mutations, the inner circle plotted the contribution (proportions) of different signatures across the patients. b) The double base signatures: original (top) and reconstructed (bottom), the top correlated DBS signatures from the COSMIC database were shown on the right. The cosine similarities were shown on the top right. c) Circus plot of the contributions of indel signatures. The outer circle plots the number of indel mutations, the inner circle plotted the contribution (proportions) of different signatures across the patients. d) Circus plot of the contributions of CNV signatures. The outer circle plots the number of

CNV mutations, the inner circle plotted the contribution (proportions) of different signatures across the patients.

a

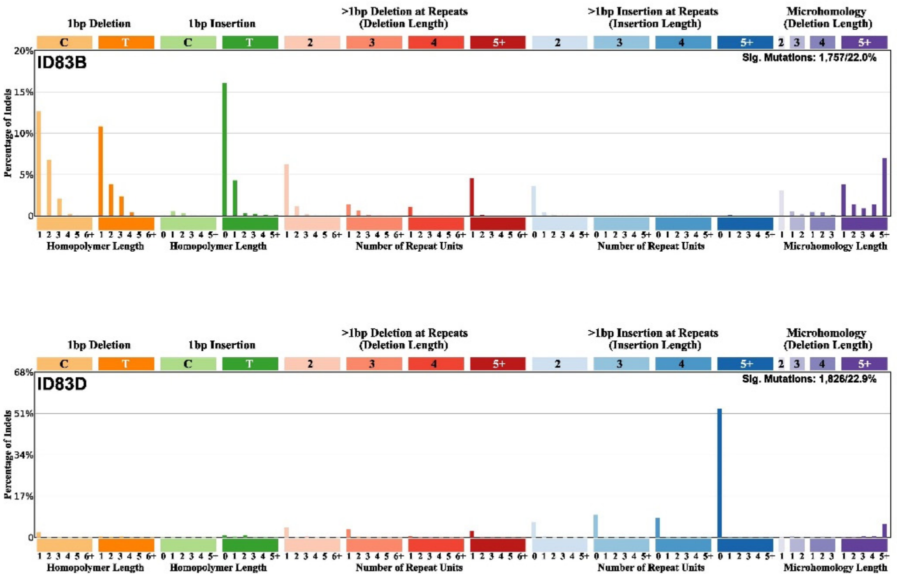

b

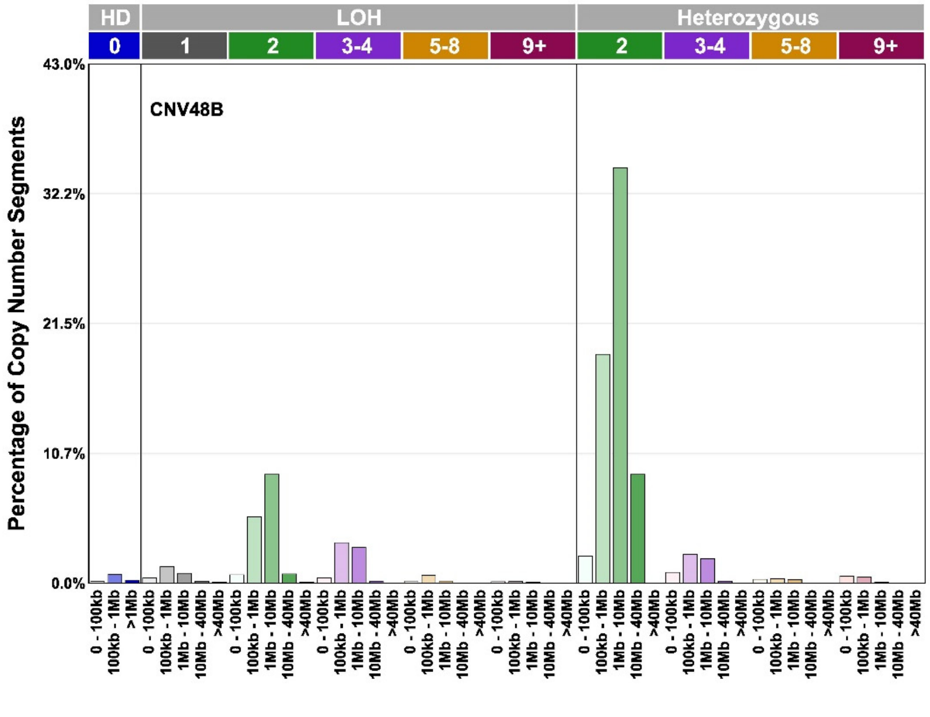

Figure S4. Novel signatures discovered in the NPC cohort. a) Two new indel signatures discovered in the NPC cohort, b) The new CNV signature discovered in the NPC cohort.

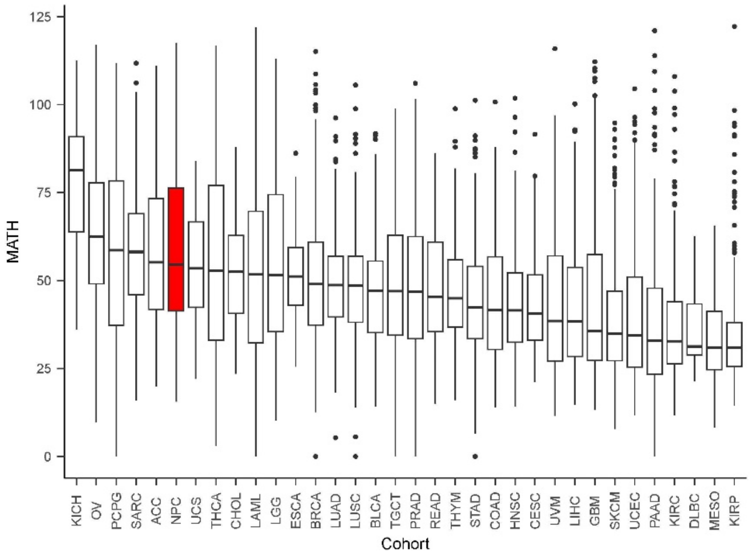

Figure S5. The boxplot of MATH score for the NPC as well as the TCGA cohort. Table S1: Reported drivers with explicit statistical evidence; Table S2: Clinical information of the cohort.
